# Healthcare costs after kidney transplantation compared to dialysis based on propensity score methods and real world longitudinal register data from Sweden

**DOI:** 10.1038/s41598-023-37814-6

**Published:** 2023-07-03

**Authors:** Ye Zhang, Ulf-G. Gerdtham, Helena Rydell, Torbjörn Lundgren, Johan Jarl

**Affiliations:** 1 Population and Development Research Center, Renmin University of China, Beijing, 100872 China; 2 School of Sociology and Population Studies, Renmin University of China, Beijing, 100872 China; 3 Health Economics Unit, Department of Clinical Sciences, Malmö, Lund University, Lund, Sweden; 4 Department of Economics, Lund University, Lund, Sweden; 5 Centre for Economic Demography, Lund University, Lund, Sweden; 6 Department of Clinical Sciences, Intervention and Technology, Karolinska Institutet, Huddinge, Sweden; 7 Swedish Renal Registry, Department of Internal Medicine, Ryhov County Hospital, Jönköping, Sweden; 8 Department of Clinical Science, Division of Transplantation Surgery, Intervention and Technology (CLINTEC), H9, Karolinska Institutet, Stockholm, Sweden

**Keywords:** Health care economics, Renal replacement therapy

## Abstract

This study aimed to estimate the healthcare costs of kidney transplantation compared with dialysis using a propensity score approach to handle potential treatment selection bias. We included 693 adult wait-listed patients who started renal replacement therapy between 1998 and 2012 in Region Skåne and Stockholm County Council in Sweden. Healthcare costs were measured as annual and monthly healthcare expenditures. In order to match the data structure of the kidney transplantation group, a hypothetical kidney transplant date of persons with dialysis were generated for each dialysis patient using the one-to-one nearest-neighbour propensity score matching method. Applying propensity score matching and inverse probability-weighted regression adjustment models, the potential outcome means and average treatment effect were estimated. The estimated healthcare costs in the first year after kidney transplantation were €57,278 (95% confidence interval (CI) €54,467–60,088) and €47,775 (95% CI €44,313–51,238) for kidney transplantation and dialysis, respectively. Thus, kidney transplantation leads to higher healthcare costs in the first year by €9,502 (*p* = 0.066) compared to dialysis. In the following two years, kidney transplantation is cost saving [€36,342 (*p* < 0.001) and €44,882 (*p* < 0.001)]. For patients with end-stage renal disease, kidney transplantation reduces healthcare costs compared with dialysis over three years after kidney transplantation, even though the healthcare costs are somewhat higher in the first year. Relating the results of existing estimates of costs and health benefits of kidney transplantation shows that kidney transplantation is clearly cost-effective compared to dialysis in Sweden.

## Introduction

End-stage renal disease (ESRD) occur when the kidneys are no longer able to function and the patient would die without renal replacement therapy, which includes dialysis and kidney transplantation (KTx)^1^. ESRD is associated with high mortality and use of healthcare resources^2^. Although patients with ESRD represent only around 0.1% of the general population, the treatment costs for them comprise 1–2% of the total healthcare expenditures in high income countries^3^. The prevalence rate of ESRD in Sweden is 980 per million inhabitants in 2018^4^ but with a total treatment costs of approximately 3.1 billion Swedish kronor (SEK) per year^5^. Kidney transplantation is the preferred treatment modality due to expected better outcomes and lower healthcare costs^5,6^. For example, Stenvinkel et al. found that dialysis costs €68,924/year while KTx costs €22,975 (€11,487) in the first (second) year^5^. Another Swedish study found that 66–79% of the expected healthcare costs over 10 years were avoided through KTx compared to dialysis, resulting in a cost savings of €380,000 (in 2012 prices) per transplanted patient^6^.

The majority of previous studies have evaluated the costs of dialysis and kidney transplantation separately^2,7^ or used a modelling approach with data collected from published aggregated estimates^7,8^. Howard et al.^7^ for example, found that increasing transplants by 10–50% would save $AUD5.8–26.2 million, while Eriksson et al.^9^ found 4–6 times higher healthcare costs for dialysis compared to KTx in Sweden. However, direct comparison of healthcare costs are likely to be biased as patients who undergo KTx are usually younger, healthier, and have higher level of education compared with patients who remain on dialysis^10^. There is a lack of cost studies accounting for this non-random treatment selection among renal replacement therapy patients, presumably because it is not possible to undertake a randomized controlled trial in the field. An alternative solution is to use observational data and an advanced propensity score approach to establish the treatment effect^11^.

The aim of this study is to compare the healthcare costs of kidney transplantation and dialysis in Sweden while adjusting for differences in patient characteristics using both the propensity score matching and the doubly robust inverse probability weighted regression adjustment approach. We will also relate the results to the health benefits (e.g., extended survival time and labour market outcomes) of KTx to comment upon the cost-effectiveness of KTx from a healthcare perspective in Sweden.

This study contributes to the existing literature on the treatment effects of different renal replacement therapy modalities on healthcare costs (costs hereafter) in three main ways. Firstly, we control for selection into different treatment modalities through propensity score matching. Secondly, we check the robustness of the results by applying the alternative inverse probability weighted regression adjustment approach. Thirdly, we present both relative and absolute estimated effects of treatments on costs. The absolute measures of treatment effects do not only provide comparative costs that can be used in further studies on economic evaluation but do also provide useful data to policy makers who need to manage high end-stage renal disease related costs.

## Materials and methods

### Data source

The data include all adults who started renal replacement therapy between 1998 and 2012, identified through the Swedish Renal Registry (SRR)^12^. Information from the Register of the Total Population (RTB)^13^, the Scandia transplant database^14^, the Longitudinal Integration Database for Health Insurance and Labour Market Studies (LISA by Swedish acronym)^15^, and regional healthcare utilization databases has been linked to the study population using the unique national personal identification number. The SRR is a register with almost 100% coverage and a data reporting incidence of 95% that includes patients’ baseline characteristics, treatment modalities, and date and cause of death^16^. The RTB includes marital status and citizenship information while LISA includes Socioeconomic Status- related data (e.g., income and education). The Scandia transplant database provides waitlist information. The regional healthcare utilization databases provide information on healthcare utilizations and costs for individuals who live in Region Skåne and Region Stockholm, two healthcare administrative areas in Sweden covering around 1/3 of the Swedish population.

### Patients characteristics

There is a lack of consensus in the literature as to which variables should be included in the propensity score model. However, the more pre-treatment covariates related to treatment assignment included, the more the selection bias is reduced. Considering covariates that were customary in previously published articles related to this topic and factors conceivably related to both healthcare costs and the choice of modality, we collected patient characteristics including age at start of renal replacement therapy, sex, year of renal replacement therapy start, income, education, marital status, citizenship, comorbidities, primary renal disease, and blood type from the linked databases. Primary renal diseases were grouped into seven categories: glomerulonephritis, adult polycystic kidney disease, diabetes mellitus, hypertension, pyelonephritis, unspecified kidney disease (unknown diagnosis), and others (known diagnosis but rare or otherwise not possible to categorize with other diagnoses). The education level, based on years of education, was categorized as mandatory education (≤ 9 years), secondary education (> 9–12 years), and higher education (> 12 years). Income was divided into quintiles, from quintile 1, the most disadvantaged quintile, to quintile 5, the most advantaged quintile.

The Charlson comorbidity index (CCI) is a simple and valid method of estimating risk of death from comorbid disease for use in longitudinal studies. It takes into account both the number and the seriousness of comorbid diseases^17^. We calculate the CCI for each patient using information in the regional healthcare utilization data based on diagnoses up to 14 years prior to start of renal replacement therapy in order to control for differences in general health condition.

### Exposures and outcomes

The primary outcome was healthcare cost, defined as total healthcare expenditures for each full year after kidney transplantation. For dialysis patients, we generated a hypothetical kidney transplant date of persons with dialysis using a propensity score matching approach (see below). As there are patients with less than 12 months of costs data following KTx due to death or censoring, and to show how costs develop during the year, we also analysed monthly costs for up to three years after KTx. The first year after KTx started from the date of KTx and lasted for 365 days. Thus, for patient that undergo KTx, the cost of the transplantation is included in the cost of the first year after KTx and the cost of re-transplantation is not included. The costs included inpatient, outpatient, and primary care cost and was adjusted to 2012 price level using the Consumer Price Index from Statistics Sweden^18^. The costs were converted to Euro (€) using 2012 average exchange rate (€1 = SEK8.7053)^19^.

### Statistical analysis

To reduce the risk of treatment selection bias, we first limited our sample to patients on the waiting list for a transplant. A previous study in Sweden have shown that the differences between patients who undergo transplantation compared to those that remain on the waiting list is smaller than if compared to those that remain on dialysis irrespective of waiting list status^10^. We can therefore assume, as is common in prior studies, that a large part of the treatment selection is removed when limiting the sample to patients on the waiting list. However, this approach cannot control for selection bias within the waiting list sample^20^. We therefore apply both the propensity-score matching (PSM) approach and the doubly robust inverse-probability-weighted regression adjustment (IPWRA) approach to further reduce the bias caused by the treatment selection. Propensity score methods allow one to mimic some of the characteristics of a randomized controlled trials in the context of an observational study. In particular, the propensity score is a balancing score, that is, conditional on the propensity score, the distribution of observed baseline covariates will be similar between treated and untreated subjects. This allows us to estimate the potential outcome mean (POM) and the average treatment effect (ATE) using observational data. The POM for kidney transplantation refers to the estimated average healthcare costs if all patients would have gotten KTx while the POM for dialysis refers to the estimated average healthcare costs if all the patients would have gotten dialysis. Thus, the ATE is the difference of the estimated average healthcare costs between KTx and dialysis over the whole sample. We adopted an intension-to-treat perspective to keep the so-called constructed randomized treatment assignment.

Wait-listed patients were included in the kidney transplantation group if they had a kidney transplant during the study period, otherwise patients were assigned to the dialysis group that includes peritoneal- and haemodialysis. The probability (propensity score) of receiving each treatment was estimated by logistic regression and used to control for systematic differences in the treatment modality groups. The weights applied in the inverse-probability-weighted regression adjustment are the inverse probability of access to KTx or dialysis. The propensity score model include the baseline patient characteristics (age at start of renal replacement therapy, sex, start year of renal replacement therapy, education, income, marital status, citizenship, primary renal disease, comorbidities (yes/no), Charlson comorbidity index, and blood type). We checked the overlap assumption that each patient has a positive probability of receiving each treatment modality. Standardized differences and variance ratios were used to assess the balance of baseline covariates between the groups before and after matching. This was done to check if the observed selection bias was reduced to acceptable levels.

For the inverse-probability-weighted regression adjustment approach, we then used the regression adjustment models (generalized linear model with log-link function and gamma distribution for cost) weighted with access to treatment. Bootstrapping with 1000 replications was used to estimate the robust standard error on which the significance tests and confidence intervals were based. For patients on the waiting list who did not undergo transplantation during the study period (i.e., patients in the dialysis group), we estimated a hypothetical kidney transplantation date in order to match the data structure of the KTx group, using a one-to-one nearest neighbour propensity score matching (NNPSM) approach. This approach paired the patients on KTx and dialysis based on their observable baseline characteristics before start of RRT^21^. The same date of KTx were assigned to both patients in the pair.

### Sensitivity analysis

Although both propensity-score matching and inverse-probability-weighted regression adjustment approaches can estimate average treatment effect and potential outcome mean, the principles of the two approaches are different. One of the drawbacks of the PSM approach is that biased estimates may be obtained if the propensity score model is mis-specified and that cases without appropriate matched controls are dropped from the analysis. Unlike PSM, the IPWRA approach provides efficient estimates by allowing the modelling of both the outcome and the treatment equations and requires that only one of the two models are correctly specified to consistently estimate the impact. It also uses the full sample. However, the PSM approach is less sensitive small sample/cell sizes and will more reliably produce estimated coefficients. We therefore use PSM as our baseline approach but test the robustness of the results using the IPWRA approach, when possible.

### Ethics approval

The study has been approved by Lund Regional Ethical Review Board (Dnr: 2014/144).

## Results

### Descriptive analysis and model assessment

Table 1 shows the baseline characteristics in the groups of patients on dialysis and kidney transplantation. Although we already limited our sample to patients on the waiting list for a transplant, there are still statistically significant different in age at start of renal replacement therapy, home county, Charlson comorbidity index, and blood type between dialysis and KTx group.

**Table 1:** Baseline characteristics in the dialysis and kidney transplantation groups (n = 693).

Baseline variable	Dialysis	Kidney transplantation	*p*
Age at start RRT, years (ref = 18–39), %	7.4	15.9	< 0.001
40–49	21.0	23.3	
50–59	36.9	36.0	
60+	34.7	24.8	
Male, %	66.5	66.6	0.9719
Education (ref = mandatory), %	32.4	27.6	0.097
Secondary school	44.3	43.2	
Higher education	23.3	29.2	
Disposable income (ref = quintile 1), %	14.2	17.3	0.3925
Quintile 2	11.9	13.6	
Quintile 3	15.9	14.0	
Quintile 4	21.6	18.8	
Quintile 5	36.4	36.3	
Marital status (ref = married), %	49.9	48.8	0.8409
Single	27.3	29.5	
Divorced	18.2	18.8	
Widowed	4.6	2.9	
Citizenship (ref = non-Swedish), %	6.8	6.3	0.7903
Swedish	93.2	93.7	
Home county§ (ref = no KTx centre), %	9.7	16.9	0.018
KTx centre	90.3	83.1	
Primary renal disease (ref = APKD ), %	10.3	17.1	0.1466
Diabetic nephropathy	35.2	16.5	
Glomerulonephritis	15.3	26.0	
Hypertension	13.1	6.6	
Pyelonephritis	2.8	3.1	
Unspecified kidney disease	6.8	9.0	
Other	16.5	21.7	
Comorbidities, %			
Hypertension	81.3	83.1	0.5651
Diabetes mellitus	43.8	20.4	< 0.001
Cardiovascular disease	31.3	17.9	< 0.001
Cancer	6.3	3.4	0.078
Blood type (ref = O), %	53.4	36.9	0.003
A	31.8	45.8	
B	11.4	12.0	
AB	3.4	5.3	
CCI, mean (SD)	3.7 (2.4)	2.6 (1.8)	< 0.001

*RRT* renal replacement therapy, *ref* reference group, *KTx* kidney transplantation, *APKD* adult polycystic kidney disease, *CCI* Charlson comorbidity index, *SD* standard deviation. ^§^Whether patient’s home county has a Tx center. Equivalized disposable income was divided into quintiles, where quintile 1 represents the most disadvantaged and quintile 5 the most advantaged.

Supplementary Table S1 online shows the baseline characteristics before and after matching in the groups of patients on dialysis and kidney transplantation. The standardized differences between the groups are generally low already from the start, following the approach of comparing patients on the waiting list. However, some differences are noted, especially in terms of age at start of renal replacement therapy, education, income and comorbidities, but the matching approach successfully handles these differences. The standardized differences are all close to zero, and the variance ratios are all close to one. We can therefore conclude that the balance of covariates between the KTx and dialysis groups is acceptable. There is no evidence that the overlap assumption is violated as the estimated density of the predicted probabilities of undergoing KTx or remaining on dialysis have most of their respective masses in the regions where they overlap (see Supplementary Fig. S1 online).

Table 2 shows the unadjusted, descriptive analysis of annual total healthcare costs for wait-listed patients over three years after kidney transplantation. Patients undergoing KTx has higher average healthcare costs in the first year after KTx, compared to patients on dialysis. However, this is turned around in the second and third year after KTx.

**Table 2:** Descriptive analysis of average healthcare costs in first to third year after kidney transplantation (€) (mean ± SD).

Healthcare costs	Dialysis	Kidney transplantation	*p*
First year after KTx (n = 693)	55,763 ± 51,197	56,281 ± 35,779	0.909
Second year after KTx (n = 600)	44,419 ± 53,100	11,299 ± 19,639	< 0.001
Third year after KTx (n = 520)	50,679 ± 52,311	11,571 ± 23,676	< 0.001

*KTx* kidney transplantation, *SD* standard deviation.

### Average treatment effect on healthcare costs

The adjusted, estimated average treatment effect are shown in Table 3. For the first year after KTx, the average cost per patient is €57,278 for patients who received a renal transplant; €9502 higher than if all patients would have received dialysis (*p* = 0.066). However, KTx is associated with €36,342 and €44,882 lower costs compared to dialysis in the second and the third year after KTx (*p* < 0.001).

**Table 3:** Average treatment effect on annual average healthcare costs of kidney transplantation compared to dialysis (€).

	Coef.	*p*	95% CI	
First year after kidney transplantation (n = 693)				
Average treatment effect				
KTx versus dialysis	9,502	0.066	-636	19,641
Potential outcome means				
KTx	57,278	< 0.001	54,467	60,088
Dialysis	47,775	< 0.001	44,313	51,238
Second year after kidney transplantation (n = 600)				
Average treatment effect				
KTx versus Dialysis	− 36,342	< 0.001	− 39,179	− 33,505
Potential outcome means				
KTx	11,513	< 0.001	9935	13,092
Dialysis	47,855	< 0.001	43,717	51,994
Third year after kidney transplantation (n = 520)				
Average treatment effect				
KTx versus Dialysis	− 44,882	< 0.001	− 61,666	− 28,097
Potential outcome means				
KTx	12,020	< 0.001	9958	14,083
Dialysis	56,902	< 0.001	52,569	61,235

*CI* confidence interval, *KTx* kidney transplantation.

**Figure 1:**
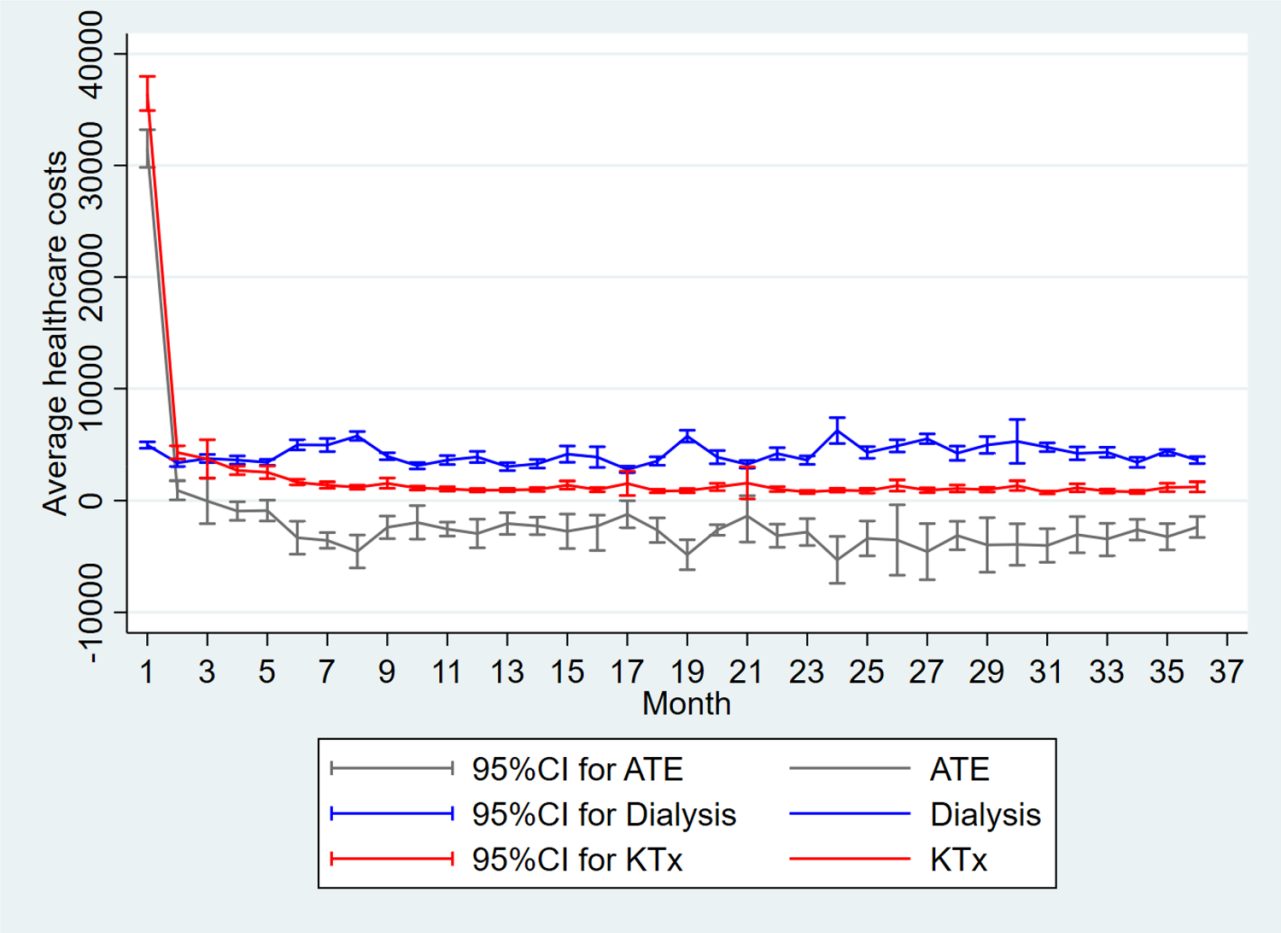
Average treatment effect on monthly average healthcare costs over three years after kidney transplantation (€). *ATE* average treatment effect (the difference in average annual healthcare costs between KTx and dialysis), *KTx* kidney transplantation.

Figure 1 shows the estimated ATE on monthly average healthcare cost for three years after KTx for wait-listed patients. The higher cost of KTx during the first year as noted in Table 3 is almost exclusively due to higher cost during the first month (ATE €31,517), i.e., presumably due to the transplantation as such and frequent check-ups. Also note that costs develop smoothly over time with less variance for KTx after the second month compared to dialysis.

### Average treatment effect on average total healthcare costs using the IPWRA approach

Table 4 shows the estimated average treatment effect on annual healthcare costs using IPWRA approach for wait-listed patients. This approach, compared to PSM, results in ATEs that reduces the positive effect of KTx compared to dialysis, but does not change the overall interpretation of the results. The analysis is thus not particularly sensitive to the treatment effect estimation approach.

**Table 4:** Sensitive analysis: average treatment effect on annual healthcare costs of kidney transplantation compared to dialysis (€).

	Coef.	*p*	95% CI	
First year after kidney transplantation (n = 693)				
Average treatment effect				
KTx versus Dialysis	13,701	0.117	− 3,422	30,823
Potential outcome means				
KTx	60,258	< 0.001	49,837	70,678
Dialysis	46,557	< 0.001	35,934	57,180
Second year after kidney transplantation (n = 600)				
Average treatment effect				
KTx versus Dialysis	− 28,865	< 0.001	− 40,896	− 16,833
Potential outcome means				
KTx	12,431	< 0.001	8,988	15,875
Dialysis	41,296	< 0.001	30,171	52,421
Third year after kidney transplantation (n = 520)				
Average treatment effect				
KTx versus Dialysis	− 38,039	< 0.001	− 54,806.1	− 21,273
Potential outcome means				
KTx	12,725	0.007	3,529	21,921
Dialysis	50,764	< 0.001	35,633	65,895

*CI* confidence interval, *KTx* kidney transplantation.

## Discussion

In this population-based study, we found that patients undergoing kidney transplantation have higher healthcare cost compared to those who had continuous dialysis in the first year after KTx, albeit borderline statistically insignificant. In the subsequent two years, KTx is associated with a significantly lower cost. When increasing the granularity, we found that KTx is associated with a higher cost compared to dialysis in the first two months after KTx, whereafter it is consistently lower with a declining trend, potentially due to reduced monitoring over time.

Comparison of healthcare costs across studies and countries should be made carefully as differences in healthcare systems, methodological approaches, and costs included in the analyses makes direct comparisons challenging. However, the results of the current study are overall consistent with prior studies, although the absolute estimates are different. We found that the annual healthcare costs per patient undergoing KTx in the first year (€57,278) were much higher than that of the second and third year (€11,513 and €12,020), which is consistent with previous studies^8,22–24^. In China, the annual medical costs of KTx were US$21,027 (€15,610) in the first year and US$14,811 (€10,996) in the second year in 2013^22^. A Dutch study found that annual healthcare costs for KTx were €85,127 in the year of transplantation only to rapidly decline in subsequent years (€29,612 and €15,018)^24^. The noticeably higher healthcare costs for KTx in the first year are mainly due to costs related to the actual transplantation (i.e., organ evaluation costs, operation costs), highlighted by the monthly costs estimations in the current study.

Our results showed that the annual healthcare costs associated with dialysis were consistently high, which is along the lines of previous studies. The above-mentioned Dutch study noted that patients on dialysis had similar healthcare costs as patients undergoing KTx in the year of KTx^24^. This can be compared to the current study where KTx is associated with higher costs compared to dialysis in the year of KTx, albeit not statistically significant. The annual healthcare costs associated with KTx in the second and third year after KTx constitutes 21–24% of the corresponding costs for dialysis in our study. This can be compared to 14–19% in the Dutch study^24^ and 21–23% in prior Swedish study^6^, indicating a dramatic cost-saving from KTx compared to dialysis from the second year.

The IPWRA results in the sensitivity analysis are very similar to the baseline PSM results. The results do not only show that our results are robust but do also suggest that our propensity score model was appropriately specified.

### Cost-effectiveness of KTx compared to dialysis

The current study indicates a €72,000 saving in healthcare costs associated with KTx compared to dialysis over the first three years after KTx. Relating these results to prior studies on the effect of KTx on other costs and outcomes will indicate the cost-effectiveness of KTx compared to dialysis.

Two important healthcare related costs of KTx are not included in the current study; costs related to procuring the graft and pharmaceuticals (especially immunosuppressants). Cost of procurement is lacking from Sweden, but a recent French study estimated this cost to €1432^25^ while a Spanish study from 2011 used $3162 (€2271)^26^. Pharmaceutical cost associated with KTx in comparison to dialysis is also lacking in Sweden. A study from Lombardy, Italy, reported the pharmaceutical cost in the first year of transplantation to €5618^27^. Although this is not the excess costs compared to dialysis, it still gives an indication of the upper bound of pharmaceutical costs associated with KTx. Applying these costs to Sweden, the cost-saving of KTx compared to dialysis falls to around €52,000 over three years.

We have previously estimated the effect of KTx on labour market outcomes compared to remaining on dialysis on the same Swedish population as in the current study. After adjusting for treatment selection, a 21 percentage points KTx advantage on the likelihood of being employed was found. This increased to 38 percentage points five years after KTx, primarily due to reduced likelihood of being employed if remaining on dialysis. In monetary terms, KTx was thus found to reduce productivity losses compared to dialysis, by €32,800 over 5 years (discounted, 2013 year’s price level)^28^.

A previous study on the same Swedish population as in the current study found a survival advantage of KTx compared with dialysis of almost 14 years after adjusting for the treatment selection bias^29^. A Kaplan–Meier survival curve shown the estimated survival time of KTx compared with dialysis (see Supplementary Fig. S2 online). Although quality of life information is lacking for the population covered in the current study, the general conclusion in the literature is that KTx is associated with higher quality of life, compared to dialysis, also after controlling for patient selection based on age and diabetes prevalence^30^. A Norwegian study noted a small but positive effect of quality of life already in the first year after KTx compared to dialysis (0.024 QALY gain)^31^.

Based on the results of the current and other studies conducted by the research team, complimented with other studies on quality of life and cost of organ procurement and pharmaceuticals, we conclude that KTx is a highly preferred treatment compared to dialysis in Sweden. KTx is both less costly and better in terms of other patient-related, sought-after outcomes.

### Strengths and limitations

This study collected population-based data from routine clinical care in the Swedish healthcare system where registered individuals have universal access. Furthermore, by using individual-level data from several nationwide registers, linked together using the personal identity number, follow-up were virtually complete regarding outcome data for inpatient, outpatient, and primary care costs, as well as data on mortality.

Even though we have limited the study sample to patients on the waiting list for transplantation and applied both propensity score matching and the inverse probability weighting approach in order to reduce the selection bias to treatment, we cannot eliminate potential bias induced by unobserved variables. However, having access to rich data material reduces the risk of bias due to unobserved factors. It should further be noted that donor healthcare costs were not included in the current study, and neither was medications (prescribed and over the counter). This will most likely lead to an underestimation of the actual cost of KTx^7^ and the cost advantage of KTx compared to dialysis would thus be lower. However, given the size of the cost saving, it seems unlikely that inclusion of these costs would change the overall conclusion of the study. Finally, the limited time frame of three post-transplant years prevents us from predicting cost levels in later years. This would be interesting given that the median (death censored) graft survival in Sweden is 23–26 years^32^ but we would expect that the cost advantage remains for as long as the graft functions.

## Conclusion

Even though kidney transplantation patients incur higher cost than dialysis in the first year after KTx, kidney transplantation is still associated with lower cost in the long run. KTx is a highly preferred treatment compared to dialysis in Sweden. Further studies are needed to assess the cost of kidney transplantation and dialysis in longer follow-up period. The findings can also be used when conducting further economic evaluation research on different types of renal replacement therapy and they give important information to health care policy makers.

## Supplementary Information

Supplementary Information.

## Data Availability

The datasets generated during and/or analysed during the current study are available from the corresponding author on reasonable request.

## References

[1] Chamberlain G (2014). The economic burden of posttransplant events in renal transplant recipients in Europe. Transplantation.

[2] Klarenbach SW (2014). Economic evaluation of dialysis therapies. Nat. Rev. Nephrol..

[3] De Vecchi AF, Dratwa M, Wiedemann ME (1999). Healthcare systems and end-stage renal disease (ESRD) therapies–an international review: costs and reimbursement/funding of ESRD therapies. Nephrol. Dial. Transplant..

[4] Swedish Renal Registry. Annual report 2019. Swedish.

[5] Stenvinkel P (2010). Chronic kidney disease: A public health priority and harbinger of premature cardiovascular disease. J. Intern Med..

[6] Jarl J (2017). Do kidney transplantations save money? A study using a before–after design and multiple register-based data from Sweden. Clin. Kidney J..

[7] Howard K (2009). The cost-effectiveness of increasing kidney transplantation and home-based dialysis. Nephrology.

[8] Villa G (2011). Cost analysis of the Spanish renal replacement therapy programme. Nephrol. Dial Transplant.

[9] Eriksson JK (2016). Healthcare costs in chronic kidney disease and renal replacement therapy: A population-based cohort study in Sweden. BMJ Open.

[10] Zhang Y (2018). Socioeconomic inequalities in the kidney transplantation process: A registry-based study in Sweden. Transplant. Direct.

[11] Curtis LH, Hammill BG, Eisenstein EL, Kramer JM, Anstrom KJ (2007). Using inverse probability-weighted estimators in comparative effectiveness analyses with observational databases. Med. Care..

[12] Swedish Renal Registry. 2016 Sep 15 [cited 2016 Sep 15]; Available from: http://www.medscinet.net/snr (2016).

[13] Register of the Total Population (RTB). Oct 19, 2017; Available from: https://www.scb.se/sv_/Vara-tjanster/Bestalla-mikrodata/Vilka-mikrodata-finns/Registret-over-totalbefolkningen-RTB/ (2017).

[14] Scandia transplant database. Oct 19, 2017; Available from: http://www.scandiatransplant.org/ (2017).

[15] Statistics Sweden (SCB). LISA database. Secondary LISA database. 2016 Sep 15; Available from: http://www.scb.se/en_/services/guidance-for-researchers-and-universities/SCB-data/longitudinalintegration-database-for-health-insurance-and-labour-market-studies-LISA-by-swedish-acronym/.eng; http://www.scb.se/lisa/swedish (2016).

[16] Schon S (2004). Renal replacement therapy in Sweden. Scand. J. Urol. Nephrol..

[17] Charlson ME, Ales KL, MacKenzie CR (1987). A new method of classifying prognostic comorbidity in longitudinal studies: Development and validation. J. Chron. Dis..

[18] Statistics Sweden (SCB)*.* 2016 Sep 15; Available from: http://www.scb.se/en_/Finding-statistics/Statistics-by-subject-area/Prices-and-Consumption/Consumer-Price-Index/Consumer-Price-Index-CPI/Aktuell-Pong/33779/Consumer-Price-Index-CPI/272151/ (2016).

[19] The Swedish Central Bank Annual average exchange rates. Jun 09, 2016; Available from: http://www.riksbank.se/sv/Rantor-och-valutakurser/Sok-rantor-och-valutakurser/?g130-SEKEURPMI=on&from=2012-01-02&to=2012-12-28&f=Year&cAverage=Average&s=Comma#search (2016).

[20] Bayat S (2010). Survival of transplanted and dialysed patients in a French region with focus on outcomes in the elderly. Nephrol. Dial Transplant..

[21] Tseng FM (2018). The impact of spousal bereavement on hospitalisations: Evidence from the Scottish Longitudinal Study. Health Econ..

[22] Zhang H (2020). Direct medical costs of end-stage kidney disease and renal replacement therapy: A cohort study in Guangzhou City, southern China. BMC Health Serv Res.

[23] Kim S-H (2017). Economic burden of chronic kidney disease in Korea using national sample cohort. J. Nephrol..

[24] Mohnen SM (2019). Healthcare costs of patients on different renal replacement modalities - Analysis of Dutch health insurance claims data. PLoS ONE.

[25] Hrifach A (2018). Organ recovery cost assessment in the French healthcare system from 2007 to 2014. Eur. J. Public Health.

[26] Domínguez J, Harrison R, Atal R (2011). Cost-benefit estimation of cadaveric kidney transplantation: The case of a developing country. Transpl. Proc..

[27] Roggeri DP (2019). Real-world data on healthcare resource consumption and costs before and after kidney transplantation. Clin. Transplant..

[28] Jarl J (2018). Effects of kidney transplantation on labor market outcomes in Sweden. Transplantation.

[29] Zhang Y (2020). Quantifying the treatment effect of kidney transplantation relative to dialysis on survival time: New results based on propensity score weighting and longitudinal observational data from Sweden. Int. J. Environ. Res. Public Health.

[30] Liem YS (2007). Quality of life assessed with the medical outcomes study short form 36-item health survey of patients on renal replacement therapy: A systematic review and meta-analysis. Value Health.

[31] Heldal K (2019). Kidney transplantation: an attractive and cost-effective alternative for older patients? A cost-utility study. Clin. Kidney J..

[32] SVENSKT NJURREGISTER ÅRSRAPPORT 2022. Available from: https://www.medscinet.net/snr/rapporterdocs/Svenskt%20Njurregister%20%C3%85rsrapport%202022%20webbversion.pdf (2022).

