# Supplementary material for: Healthcare costs after kidney transplantation compared to dialysis based on propensity score methods and real world longitudinal register data from Sweden
Source: Sci Rep. 2023 Jul 3;13:10730. doi: 10.1038/s41598-023-37814-6 (PMC10318027; doi:10.1038/s41598-023-37814-6)

**Supplementary Information**

Supplementary Table S1. Baseline characteristics before and after matching in the dialysis and kidney transplantation groups

| Baseline variable | Standardized differences | | Variance ratio | |
| --- | --- | --- | --- | --- |
|  | Raw | Matched | Raw | Matched |
| Age at start RRT, years  (ref=18–39) |  |  |  |  |
| 40–49 | .012 | .225 | 1.004 | 1.433 |
| 50–59 | -.016 | -.383 | .980 | .951 |
| 60+ | -.248 | .014 | .783 | 1.017 |
| Male, % | .067 | .311 | .950 | .899 |
| Education  (ref= mandatory) |  |  |  |  |
| Secondary school | .026 | -.012 | .996 | .997 |
| Higher education | .111 | .062 | 1.111 | 1.066 |
| Disposable income  (ref= quintile 1) |  |  |  |  |
| Quintile 2 | .048 | .047 | 1.095 | 1.106 |
| Quintile 3 | -.030 | -.025 | .931 | .951 |
| Quintile 4 | .003 | -.332 | .993 | .703 |
| Quintile 5 | -.026 | .222 | .973 | 1.22 |
| Marital status  (ref = married) |  |  |  |  |
| Single | .118 | .202 | 1.118 | 1.274 |
| Divorced | .104 | -.333 | 1.185 | .690 |
| Widowed | -.032 | .061 | .841 | 1.423 |
| Citizenship  (ref = non-Swedish) |  |  |  |  |
| Swedish | .099 | -.177 | .719 | 2.211 |
| Home county^§^  (ref = no KTx centre) |  |  |  |  |
| KTx centre | -.490 | -.213 | 5.475 | 1.690 |
| Primary renal disease  (ref= APKD ) |  |  |  |  |
| Diabetic nephropathy | -.365 | -.041 | .628 | .933 |
| Glomerulonephritis | .307 | -.272 | 1.584 | .806 |
| Hypertension | -.209 | -.161 | .520 | .610 |
| Pyelonephritis | .141 | .199 | 2.577 | 6.817 |
| Unspecified kidney disease | -.001 | .0404 | .986 | 1.124 |
| Other | .027 | .197 | 1.028 | 1.397 |
| Comorbidities |  |  |  |  |
| Hypertension | -.028 | -.092 | 1.038 | 1.178 |
| Diabetes mellitus | -.450 | -.058 | .670 | .929 |
| Cardiovascular disease | -.320 | .138 | .662 | 1.277 |
| Cancer | -.156 | -.024 | .493 | .879 |
| Blood type  (ref=O) |  |  |  |  |
| A | .190 | .157 | 1.076 | 1.075 |
| B | .0153 | -.013 | 1.025 | .972 |
| AB | .144 | -.460 | 2.010 | .296 |
| CCI | -.758 | -.106 | .725 | 1.235 |

RRT=renal replacement therapy; ref = reference group; KTx = kidney transplantation; APKD = adult polycystic kidney disease; CCI = Charlson comorbidity index.  ^§^Whether patient’s home county has a Tx center. Equivalized disposable income was divided into quintiles, where quintile 1 represents the most disadvantaged and quintile 5 the most advantaged.

Supplementary Figure S1. Overlap plot by treatment groups – probability of being in the dialysis group

0

2

4

6

Density

0

.2

.4

.6

.8

Propensity score of dialysis

Dialysis

Kidney transplantation

Supplementary Figure S2. Kaplan-Meier survival curve by treatment groups


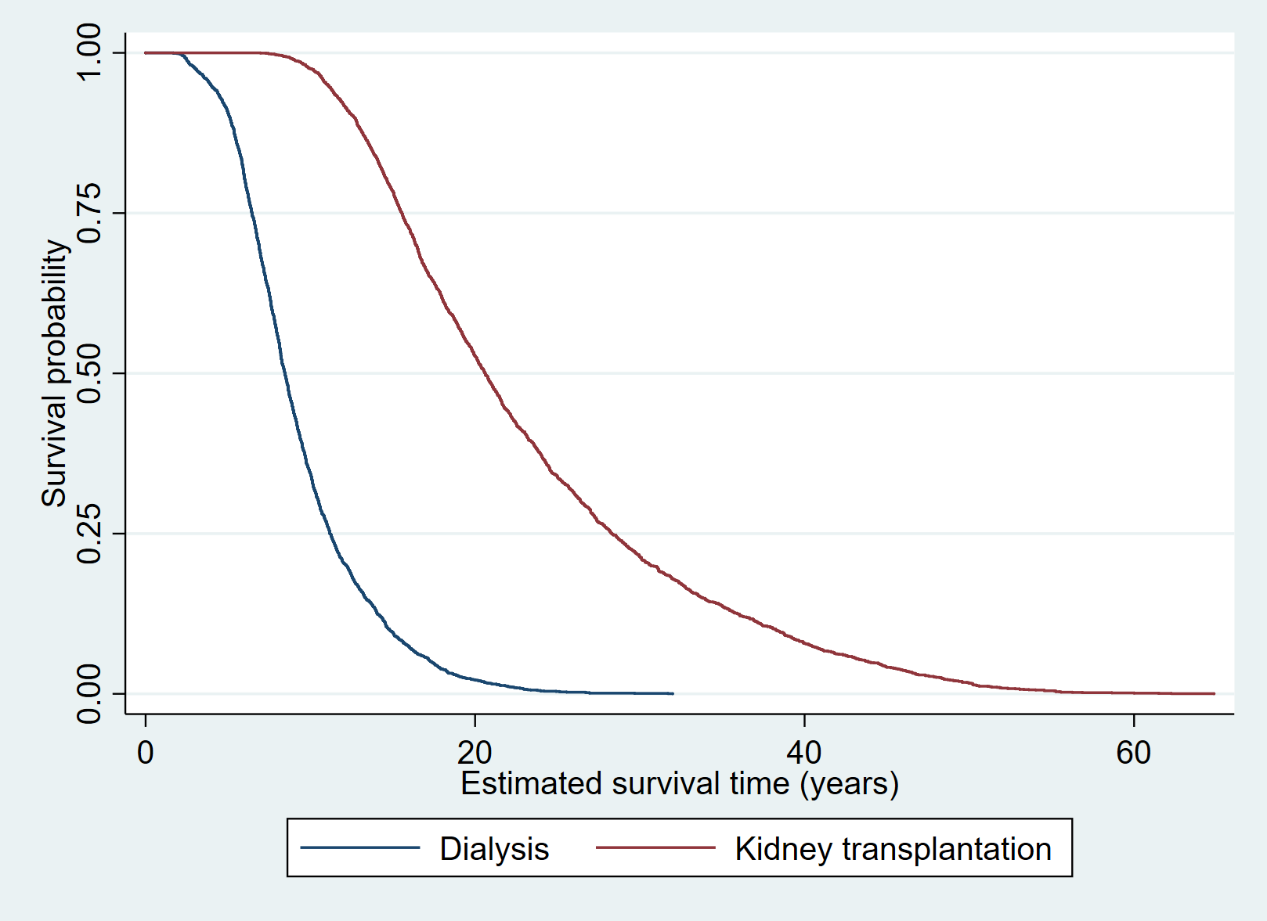

Supplement: Supplementary file 1 — Supplementary Information. [file 41598_2023_37814_MOESM1_ESM.docx]
